# Required coefficient of friction in the anteroposterior and mediolateral direction during turning at different walking speeds

**DOI:** 10.1371/journal.pone.0179817

**Published:** 2017-06-22

**Authors:** Takeshi Yamaguchi, Akito Suzuki, Kazuo Hokkirigawa

**Affiliations:** 1 Graduate School of Engineering, Tohoku University, Sendai, Miyagi, Japan; 2 Graduate School of Bioengineering, Tohoku University, Sendai, Miyagi, Japan; West Virginia University, UNITED STATES

## Abstract

This study investigated the required coefficient of friction (RCOF) and the tangent of center of mass (COM)–center of pressure (COP) angle in the mediolateral (ML) and anteroposterior (AP) directions during turning at different walking speeds. Sixteen healthy young adults (8 males and 8 females) participated in this study. The participants were instructed to conduct trials of straight walking and 90° step and spin turns to the right at each of three self-selected speeds (slow, normal, and fast). The ML and AP directions during turning gait were defined using the orientation of the pelvis to construct a body-fixed reference frame. The RCOF values and COM–COP angle tangent in the ML direction during turning at weight acceptance phase were higher than those during straight walking, and those values increased with increasing walking speed. The ML component of the RCOF and COM–COP tangent values during weight acceptance for step turns were higher than those for spin turns. The mean centripetal force during turning tended to increase with an increase in walking speed and had a strong positive correlation with the RCOF values in the ML direction (R = 0.97 during the weight acceptance phase; R = 0.95 during the push-off phase). Therefore, turning, particularly step turn, is likely to cause lateral slip at weight acceptance because of the increased centripetal force compared with straight walking. Future work should test at-risk population and compare with the present results.

## Introduction

Slip-induced falls are the most frequent events leading to injury at home [1,2] and workplaces [3,4]. During walking, the tangential force applied to the floor cannot exceed the friction force. Thus, the ratio of the tangential force to the vertical force applied to the floor, i.e., traction coefficient, must be lower than the friction coefficient at the shoe–floor interface during the stance phase. The traction coefficient is calculated by dividing the horizontal ground reaction force (GRF) by the vertical GRF. The peak traction coefficients observed just after heel contact [5] and just before toe-off [6] are recognized as the coefficients of friction (RCOF_h_, RCOF_t_) required to prevent slips during the braking and propulsion phases, respectively.

Majority of the research on slips and falls have investigated straight gait, and many RCOF values have been reported for straight gait [7–11]. In daily life, turns and non-straight steps commonly occur in addition to straight gait [12]. However, only a few research studies regarding slips and falls during turning have been conducted and only a few have investigated RCOF values during turning [13–15]. Turning requires greater RCOF values to prevent slips [13, 16] and RCOF values increased with an increase in walking speed during turning [13]; thus, turning has a higher probability of slips than straight walking and turning at fast speed is more hazardous.

Center of mass (COM)–center of pressure (COP) angle tangent exhibits strong positive correlation with RCOF values for straight walking, turning, gait termination, and initiation [11, 15]. The COM–COP angle is defined as the angle between the line connecting COM and COP and the vertical line passing through COM. Thus, increased COM–COP angle increases RCOF values. While turning, the centrifugal force acts on COM and individuals lean in toward the apex (toward the center of rotation) to compensate for the centripetal force created when turning against the centrifugal force [17]. Therefore, the COM–COP angle in the mediolateral (ML) direction during turning, i.e., the lean angle of the body in the radial direction, is larger than that during straight gait, which will result in an increased traction coefficient in the ML direction during turning. The increased ML component of the RCOF values during turning will increase the RCOF values compared with straight walking if the anteroposterior (AP) component of the RCOF values is not different. Because the centripetal force is proportional to the velocity squared and inverse of the turning radius, the lean angle of the body (COM–COP angle) in the ML direction will be affected by turning speed [18] and turning radius [19]. Thus, an increase in turning speed could increase the ML component of the COM-COP angle and the RCOF values. Whether or not slip occurs is determined by the relationship between the friction coefficient at the shoe–floor interface and the magnitude of the RCOF value. However, even if the magnitude of RCOF value is the same, the direction of slipping is affected by the ratio of ML and AP components of the RCOF values. Yamaguchi et al.[15] found that slips occur more laterally with respect to the direction of progression during turning, which could be due to an increase in the contribution of the ML component with respect to the AP component in the RCOF values. However, to date, there is no research investigating the RCOF values in the ML and AP directions during turning.

Turning can be classified into two strategies: spin turns (ipsilateral turns) and step turns [20, 21]. The step turn involves a change in the direction opposite to the stance limb whereas the spin turn involves a change in the direction toward the same side as the stance limb. Fino and Lockhart [13] indicated that there were no significant differences in the resultant RCOF values between step and spin turns during turning. However, the center of mass trajectory with respect to COP of supporting foot during step turns and spin turns is different [21]. Fino and Lockhart [14] demonstrated that the turning radius differs among these two turning strategies. Thus, the ML component of the RCOF values and COM-COP angle will be affected by the turning strategies.

Therefore, we investigated the RCOF values and COM–COP angle tangent in the ML and AP directions during turning at different walking speeds. We hypothesized the following: 1) the ML component of the RCOF values and the COM–COP angle tangent during turning will be larger than those during straight walking, which will contribute to the increase in the resultant RCOF values during turning; 2) while turning, those variables will increase with an increase in turning speed due to an increase in the centripetal force; and 3) those variables are different among step and spin turns.

## Methods

### Subjects

This study included eight young adult males and eight young adult females aged 21.4 ± 1.2 years (range: 20–23 years), 1.65 ± 0.08 m in height (range: 1.56–1.77 m), and weighing 60.1 ± 7.4 kg (range: 51–79 kg). Participants were informed of the protocol and gave written informed consent prior to the experiment. The protocol was approved by the Institutional Review Board of Tohoku University.

### Experimental procedure

**Fig 1:**
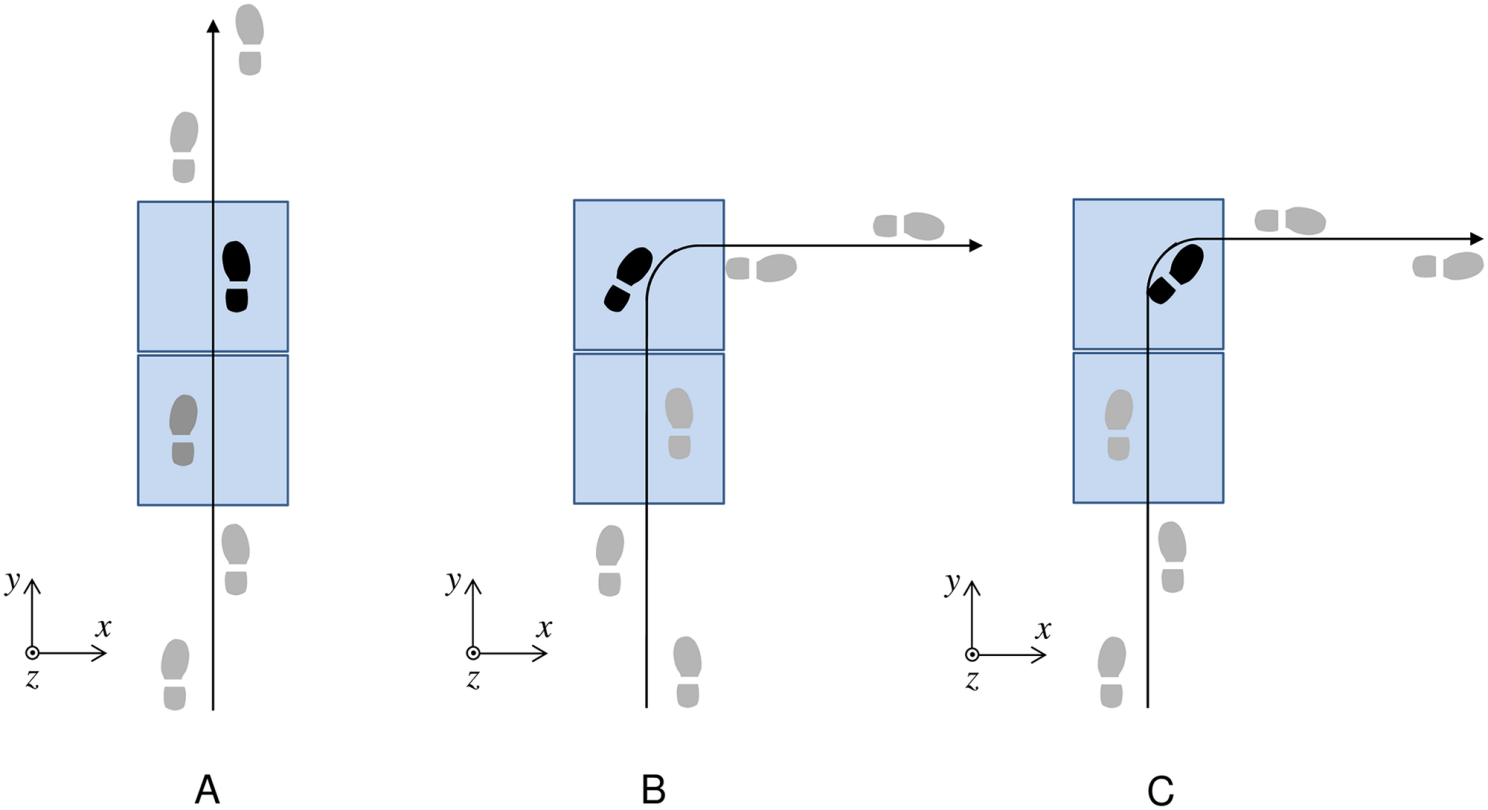
Schematic footprints of (A) straight gait, (B) 90° step turn to the right, and (C) 90° spin turn to the right. The shaded area represents the force plate embedded in the walkway. Steps not analyzed are shown in grey.

Fig 1 shows schematic diagrams of the instructions given to subjects. The *x*- and *y*-coordinates were set as the AP and ML directions, respectively, during straight walking. Gait trials were performed on a 5 m-long walkway. Two force plates (MG-2060, Anima Corp., Tokyo, Japan) were installed approximately 2 m from the start position for collecting GRFs. An eight-camera motion-capture system (MA8000, Anima Corp., Tokyo, Japan) measured full body kinematics from 16 infrared-reflective markers placed bilaterally over the acromiale, epicondylus lateralis of humerus, stylion ulnare, spina iliaca anterior superior, trochanter, epicondylus lateralis of femur, sphyrion fibulare, and fifth metatarsal heads. GRFs and 3D motion data were collected at a sampling rate of 500 Hz. The participants were provided with commercially available walking shoes (EASYSTAR2, Mizuno Corporation, Osaka, Japan).

The participants were instructed to conduct straight walking and 90° step and spin turns to the right at each of three self-selected speeds (slow, normal, and fast). Thus, the gait trials comprised nine blocks, i.e., three straight walking blocks at three different speeds, three 90° step turns at three different speeds, and three 90° spin turns at three different speeds. Sedgman et al. [22] reported that the angle of turning in daily life is between 76° and 120°. Thus, we used 90° turn trials in this study. Step turns involved turning to the right with the left foot as the pivot foot, whereas spin turns involved turning to the right with the right foot as the pivot foot [21]. For the low and high speed trials, participants were asked to walk at a pace slower than their normal walking speed and a pace faster than their normal walking speed, respectively. In the straight walking trial, they were instructed to walk in a straight line, landing on each force plate with each of their feet [Fig 1A]. They were allowed to step with either the left or right foot on each force plate. In the turning trials, they were instructed to walk in a straight line and turn 90° to the right with the left [step turn, Fig 1B] or right foot [spin turn, Fig 1C], landing on the second force plate. Lines were marked on the floor to indicate the turning direction. Participants were given a practice period to become accustomed to walking straight, stepping, and spinning at each of the three self-selected speeds. The starting position (approximately 2 m from the 1^st^ force plate) was adjusted so that foot strike occurred on the force plate. The order of each block of trials was randomized to eliminate any order effect. Each trial was replicated five times under the same conditions (45 trials per subject).

### Data analysis

Kinetic and kinematic data were collected from each trial; thus, 720 trials (45 trials × sixteen participants) were analyzed. GRF data (*F*_x_, *F*_y_, and *F*_z_) for the 2^nd^ force plate were collected and used to calculate COP with motion analysis software (MA8000, Anima Corp., Tokyo, Japan). The whole body COM behavior (position and velocity) was estimated using a seven-segment rigid link model (bilateral feet, shanks, and thighs, and trunk) from kinematic data with the motion analysis software. The GRF data and kinematic data were low-pass filtered with a cut-off frequency of 10 Hz using a fourth-order, zero-lag, Butterworth filter. The subsequent analyses were conducted using Matlab (Mathworks Inc., Massachusetts, USA).

#### Approach speed

The approach speed was defined as the speed of the participant before the foot touched the 2^nd^ force plate. It was calculated using the average velocity of COM in the *y* direction over 200 ms before the vertical GRF of the 2^nd^ force plate was detected.

#### Centripetal force

The centripetal force *F*_c_ applied to COM during turning was computed using the turning speed *v*_COM_ and turning radius of COM *r*_COM_, as follows:

$$F_{c}=\frac{mv_{COM}^{2}}{r_{COM}}$$ (1)

where *m* was the whole body mass. The turning speed was defined as the average velocity of COM during the stance phase of the pivoting foot, which landed on the 2^nd^ force plate. The curvature of the COM trajectory during turning was calculated using a least-square quadratic fit to the COM trajectory in the *x–y* plane, and the turning radius was calculated as the inverse of the curvature [14].

#### Traction coefficient and required coefficient of friction

The traction coefficient was defined as the ratio between the horizontal GRF and vertical GRF *F*_h_/*F*_z_. The horizontal GRF was calculated as follows:

$$F_{h}=\sqrt{F_{x}^{2}+F_{y}^{2}}$$ (2)

RCOF_h_ and RCOF_t_ were defined as the maximum peak value of the traction coefficient between 5% and 50% of the stance phase [23] and between 50% and 100% of the stance phase, respectively.

**Fig 2:**
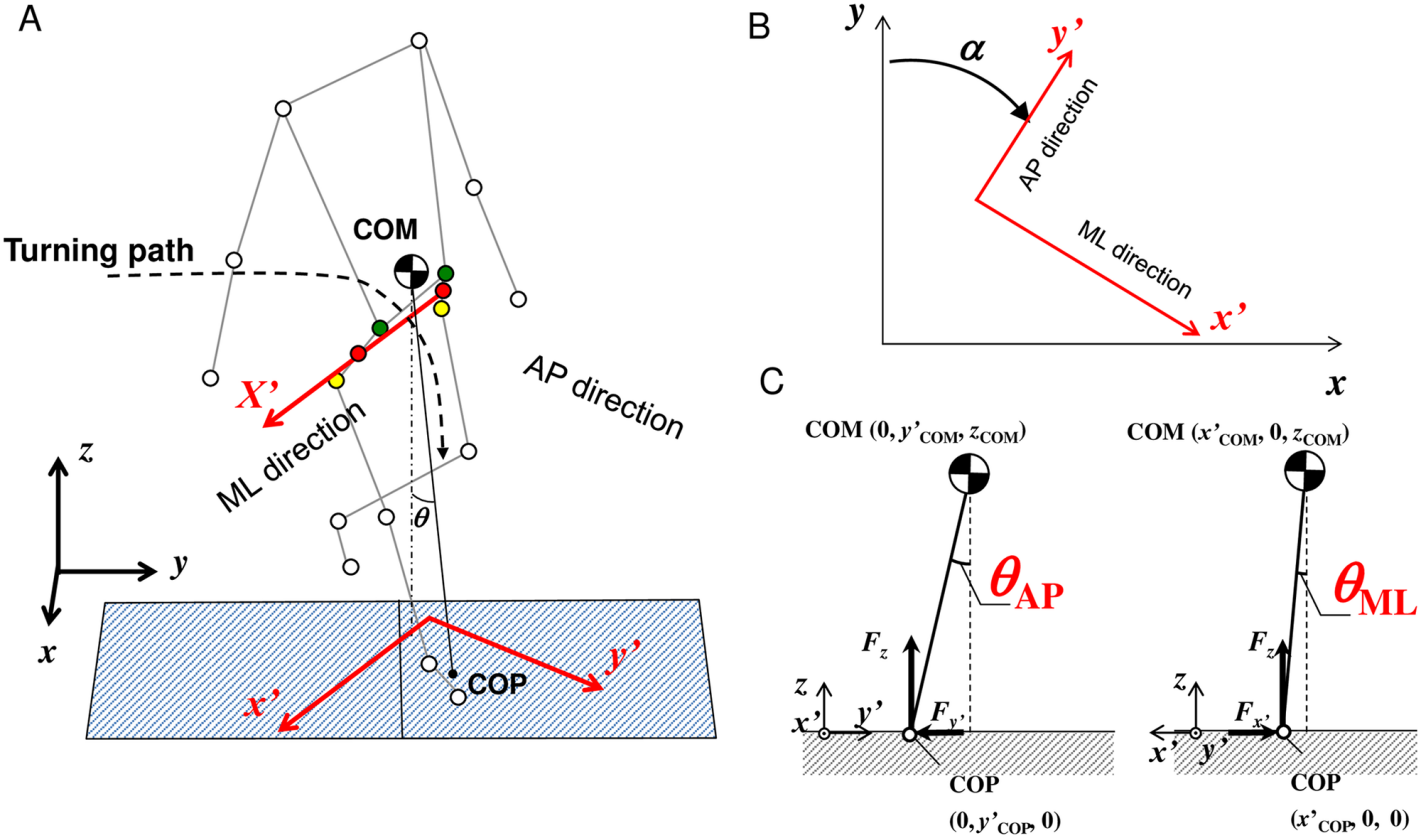
Schematic diagram of (A) the coordinate system of the reference frame (*x′*–*y′*–*z*), (B) the pelvis rotation angle *α*, and (C) COM–COP angles in AP (*θ*_AP_) and ML (*θ*_ML_) directions during turning. Note that Fig 2A is an example of spin turn. The orientation of the pelvis was used to define the body reference frame using the iliac crest (green) and trochanter (yellow) markers on each side of the body. The reference frame was defined by the projection of the body reference frame onto the global x-y plane. The red circles represent the mean position of the left/right iliac crest and left/right trochanter markers. The pelvis orientation angle *α* represents the angle between the body and global references. *X*′ and *y*′ represent the ML direction and AP direction, respectively.

In the straight walking trial, *x*- and *y*-coordinates respectively represent the ML and AP directions; thus, the traction coefficient in the ML and AP directions were calculated as *F*_x_/*F*_z_ and *F*_y_/*F*_z_, respectively. On the other hand, in the turning trial, the ML and AP directions were defined using the orientation of the pelvis to construct a body-fixed reference frame [14, 24] as shown in Fig 2A. As shown in Fig 2A, the body-fixed frame *X*′ was constructed as the line through the mean position of the left iliac crest and left trochanter markers as the origin and the mean position of the right iliac crest and right trochanter markers. The reference frame *x*′ was defined by the projection of the *X*′ axis onto the *x*-*y* plane and the *y*′ was defined as the line perpendicular to the *x*′ axis on the *x*-*y* plane as shown in Fig 2A. Thus, the *x′* axis was the ML direction and the *y*′ axis was the AP direction during turning trials. The horizontal GRFs were rotated about the vertical axis to each local coordinate system using the angles between the global coordinate system and local coordinate system, which corresponded to the pelvis rotation angle *α* [Fig 2B], as follows:

$$\left[\begin{array}{l} \begin{array}{l} F_{x\prime }\\ F_{y}{}_{\prime } \end{array} \end{array}\right]=\left[\begin{array}{ll} \text{cos}\alpha & \text{sin}\alpha \\ -\text{sin}\alpha & \text{cos}\alpha \end{array}\right]\left[\begin{array}{l} \begin{array}{l} F_{x}\\ F_{y} \end{array} \end{array}\right]$$ (3)

where *F*_x′_ and *F*_y′_ were horizontal GRFs in the ML and AP directions, respectively, during turning [Fig 2C]. Therefore, the traction coefficient in the ML and AP directions in the turning trials were calculated as *F*_x′_/*F*_z_ and *F*_y′_/*F*_z_, respectively. The RCOF_h_ and RCOF_t_ values in the ML and AP directions (RCOF_h_ML_, RCOF_h_AP_, RCOF_t_ML_, and RCOF_t_AP_, respectively) were the values of the traction coefficient in the ML and AP directions at the instance of RCOF_h_ and RCOF_t_, respectively.

#### COM–COP angle tangent

The COM–COP angle *θ* was calculated using COM, COP, and the vertical projection of COM on the floor as follows:

$$\theta =\text{atan}\left(\frac{\sqrt{{\text{(}x_{\text{COP}}-x_{\text{COM}}\text{)}}^{\text{2}}+{\text{(}y_{\text{COP}}-y_{\text{COM}}\text{)}}^{2}}}{z_{\text{COM}}}\right)$$ (4)

where *x*_COM_, *y*_COM_, and *z*_COM_ were *x*, *y*, and *z* coordinates of COM, and *x*_COP_ and *y*_COP_ were the *x* and *y* coordinates of the COP of the supporting foot (on the 2^nd^ force plate). The COM–COP angle in the ML and AP directions were calculated as follows:

$$\theta_{\text{ML}}=\{\begin{array}{ll} \text{atan}\left(\frac{x_{\text{COP}}-x_{\text{COM}}}{z_{\text{COM}}}\right) & \text{for straight walking}\\ \text{atan}\left(\frac{x\prime_{\text{COP}}-x\prime_{\text{COM}}}{z_{\text{COM}}}\right) & \text{for turning trial} \end{array}$$ (5)

$$\theta_{\text{A}P}=\{\begin{array}{ll} \text{atan}\left(\frac{y_{\text{COP}}-y_{\text{COM}}}{z_{\text{COM}}}\right) & \text{for straight walking}\\ \text{atan}\left(\frac{y\prime_{\text{COP}}-y\prime_{\text{COM}}}{z_{\text{COM}}}\right) & \text{for turning trial} \end{array}$$ (6)

where *x*′_COM_, *y*′_COM_, and *z*_COM_ were *x*′, *y*′, and *z* coordinates of COM, and *x*′_COP_ and *y*′_COP_, were the *x*′ and *y*′ coordinates of COP of the supporting foot [on the 2^nd^ force plate, Fig 2C]. The values of tan*θ*_h_ and tan*θ*_t_ were the values of tan*θ* at the instance of RCOF_h_ and RCOF_t_, respectively. The values of tan*θ*_h_ML_, tan*θ*_h_AP_, tan*θ*_t_ML_, and tan*θ*_t_AP_ were the values of tan*θ* in the ML and AP directions at the instance of RCOF_h_ and RCOF_t_, respectively. Note that in the straight walking trials, for comprehensibility, the GRF and kinematic data in the *x*-direction was multiplied by −1 when the left foot landed on the 2^nd^ force plate so that the GRF and kinematic data were adjusted as the right foot landed on the 2^nd^ force plate.

#### Statistical analysis

Statistical analysis was performed using SPSS Statistics for Windows Version 19.0 (IBM Corp., Armonk, NY, USA). Two-way repeated-measures analysis of variance (ANOVA) was used to test if the mean values of approach speed, RCOF_h_, RCOF_t_, RCOF_h_ML_, RCOF_h_AP_, RCOF_t_ML_, RCOF_t_AP_, tan*θ*_h_, tan*θ*_t_, tan*θ*_h_ML_, tan*θ*_h_AP_, tan*θ*_t_ML_, and tan*θ*_t_AP_ were affected by walking speed (slow, normal, and fast) and trial types (straight walking, step turn, and spin turn). A two-way repeated-measures ANOVA was also performed to test if the mean values of *r*_COM_, *v*_COM_, and *F*_c_ were affected by walking speed (slow, normal, and fast) and turning strategy (step and spin turns). A post-hoc paired t-test with a Bonferroni correction was used to determine specific significant differences between walking speed and trial type or turning strategy. A bivariate regression analysis between RCOF-related parameters (RCOF_h_, RCOF_t_, RCOF_h_ML_, RCOF_h_AP_, RCOF_t_ML_, and RCOF_t_AP_) and tan*θ*-related parameters (tan*θ*_h_, tan*θ*_t_, tan*θ*_h_ML_, tan*θ*_h_AP_, tan*θ*_t_ML_, and tan*θ*_t_AP_) was performed by grouping all trial conditions to investigate the correlation between them. A bivariate regression analysis between centripetal force *F*_c_ and RCOF values for turning trials in the ML direction (RCOF_h_ML_, RCOF_t_ML_) was also performed whether the increase in the value of *F*_c_ increased the RCOF_h_ML_ and RCOF_t_ML_. The significance level was set at p = 0.05.

## Results

### Approach speed, turning radius, turning speed, and centripetal force

As shown in Table 1, mean approach speeds increased with an increase in walking speed (*p* < 0.001) for each trial type. There were no significant differences in approach speed between the step and spin turns (p > 0.05). As shown in Table 2, the centripetal force for step turns at fast speed was larger than that at slow speed (p < 0.001), and the centripetal force for spin turns at normal and fast speeds were larger than those at slow speeds (p < 0.01). The centripetal force for step turns was larger than that for spin turns at slow and normal speeds (p < 0.05).

**Table 1:** Mean approaching speed (SD).

Trial type	Slow	Normal	Fast
Straight walking	1.08 (0.13)^c^^,^^d^	1.26 (0.13)^a^^,^^c^^,^^e^	1.49 (0.14)^a^^,^^b^^,^^d^^,^^e^
Step turn	1.03 (0.14)^c^^,^^d^	1.19 (0.15)^a^^,^^c^^,^^e^	1.37 (0.12)^a^^,^^d^^,^^e^
Spin turn	1.08 (0.11)^c^^,^^d^	1.21 (0.13)^c^^,^^e^	1.38 (0.08)^b^^,^^d^^,^^e^

Significant differences, p < 0.05:

^a^ between straight walking and step turn;

^b^ between straight walking and spin turn;

^c^ slow and normal speed conditions;

^d^ between slow and fast speed conditions;

^e^ between normal and fast speed conditions

**Table 2:** Mean turning radius, turning speed, and centripetal force during turning (SD).

Trial type	*r*_COM_ (m)			*v*_COM_ (m/s)			*F*_c_ (N)		
	Slow	Normal	Fast	Slow	Normal	Fast	Slow	Normal	Fast
Step turn	0.21^a^^,^^c^ (0.08)	0.23^a^^,^^d^ (0.08)	0.28^a^^,^^c^^,^^d^ (0.09)	0.87^a^^,^^b^^,^^c^ (0.11)	0.99^a^^,^^b^^,^^d^ (0.10)	1.14^a^^,^^c^^,^^d^ (0.10)	250.7^a^^,^^c^ (94.65)	287.8^a^ (107.7)	307.0^c^ (94.64)
Spin turn	0.31^a^^,^^c^ (0.11)	0.33^a^^,^^d^ (0.11)	0.36^a^^,^^c^^,^^d^ (0.13)	0.95^a^^,^^b^^,^^c^ (0.09)	1.07^a^^,^^b^^,^^d^ (0.10)	1.20^a^^,^^c^^,^^d^ (0.09)	201.9^a^^,^^b^^,^^c^ (90.92)	245.0^a^^,^^b^ (122.0)	270.3^c^ (108.2)

Significant differences, *p* < 0.05:

^a^ between step and spin turns;

^b^ between slow and normal speed conditions;

^c^ between slow and fast speed conditions;

^d^ between normal and fast speed conditions

### Pelvis rotation angle

**Fig 3:**
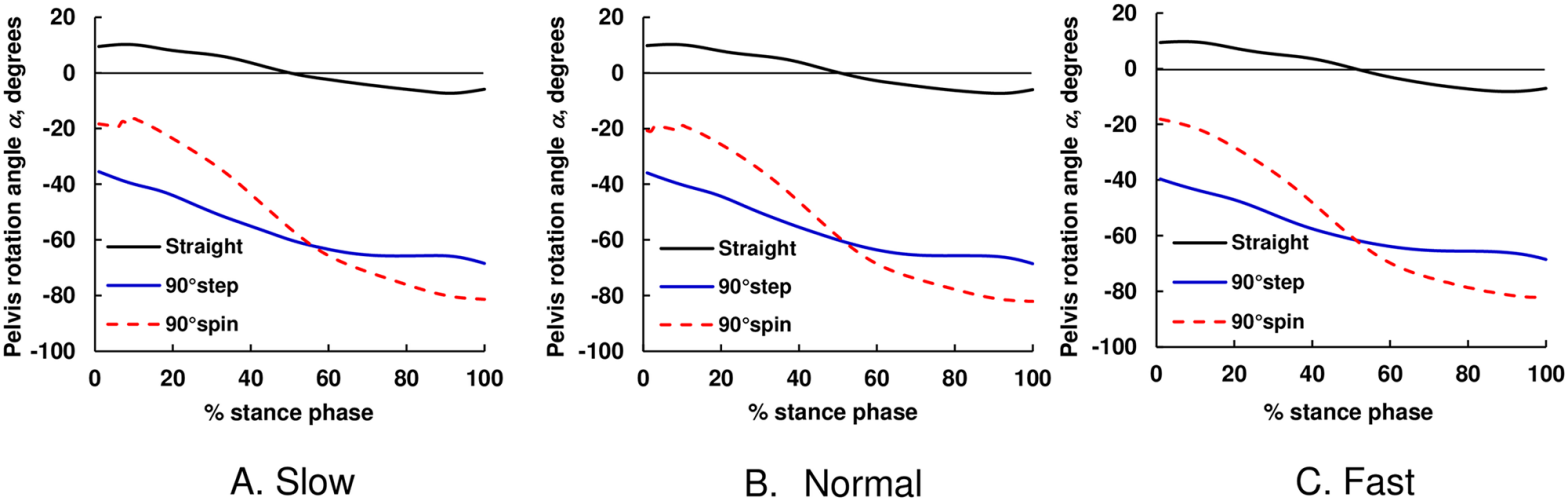
The average temporal pattern of the mean pelvis orientation angle during straight walking and turning. The black solid line shows straight walking, blue solid line shows 90° step turns, and red dashed line shows 90° spin turns. Note that the pelvis rotation angle for straight walking trials was adjusted for the right foot landing on the 2^nd^ force plate. (A) Slow, (B) Normal, and (C) Fast.

As shown in Fig 3, the mean pelvis rotation angle changed from 10° to −10° for straight walking at all speeds. The magnitude of the mean pelvis rotation angle for spin turns was significantly increased with an increase in the percentage of stance phase compared with step turns, and the mean pelvis rotation angle for spin turns exceeded that of step turns after 50% of the stance phase for all speeds.

### Temporal pattern of traction coefficient and COM–COP angle tangent

**Fig 4:**
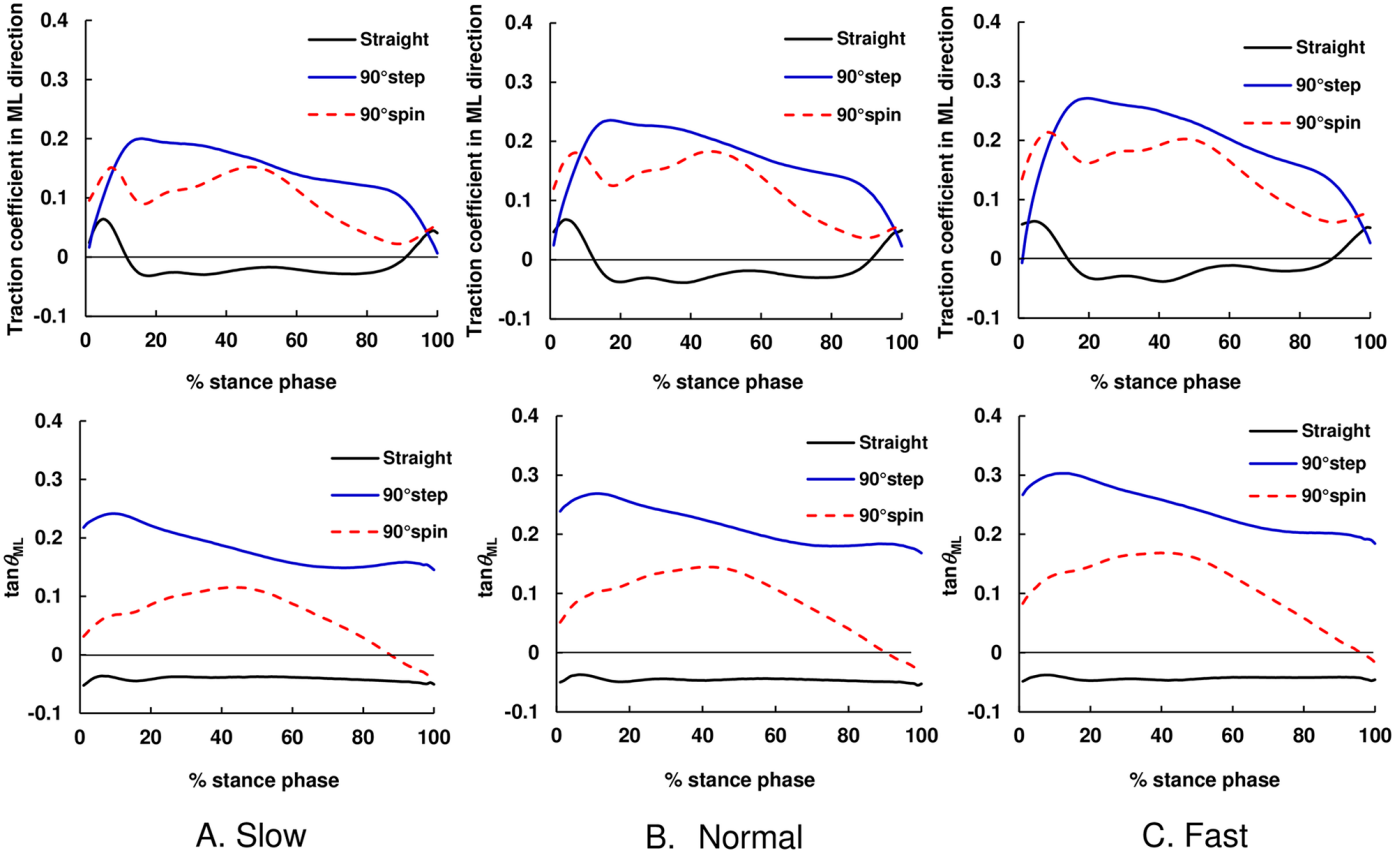
The average temporal pattern of mean values of traction coefficient and tangent of the COM–COP angle in the ML direction during straight walking and turning. The black solid line shows straight walking, blue solid line shows 90° step turns, and red dashed line shows 90° spin turns. (A) Slow, (B) Normal, and (C) Fast.

**Fig 5:**
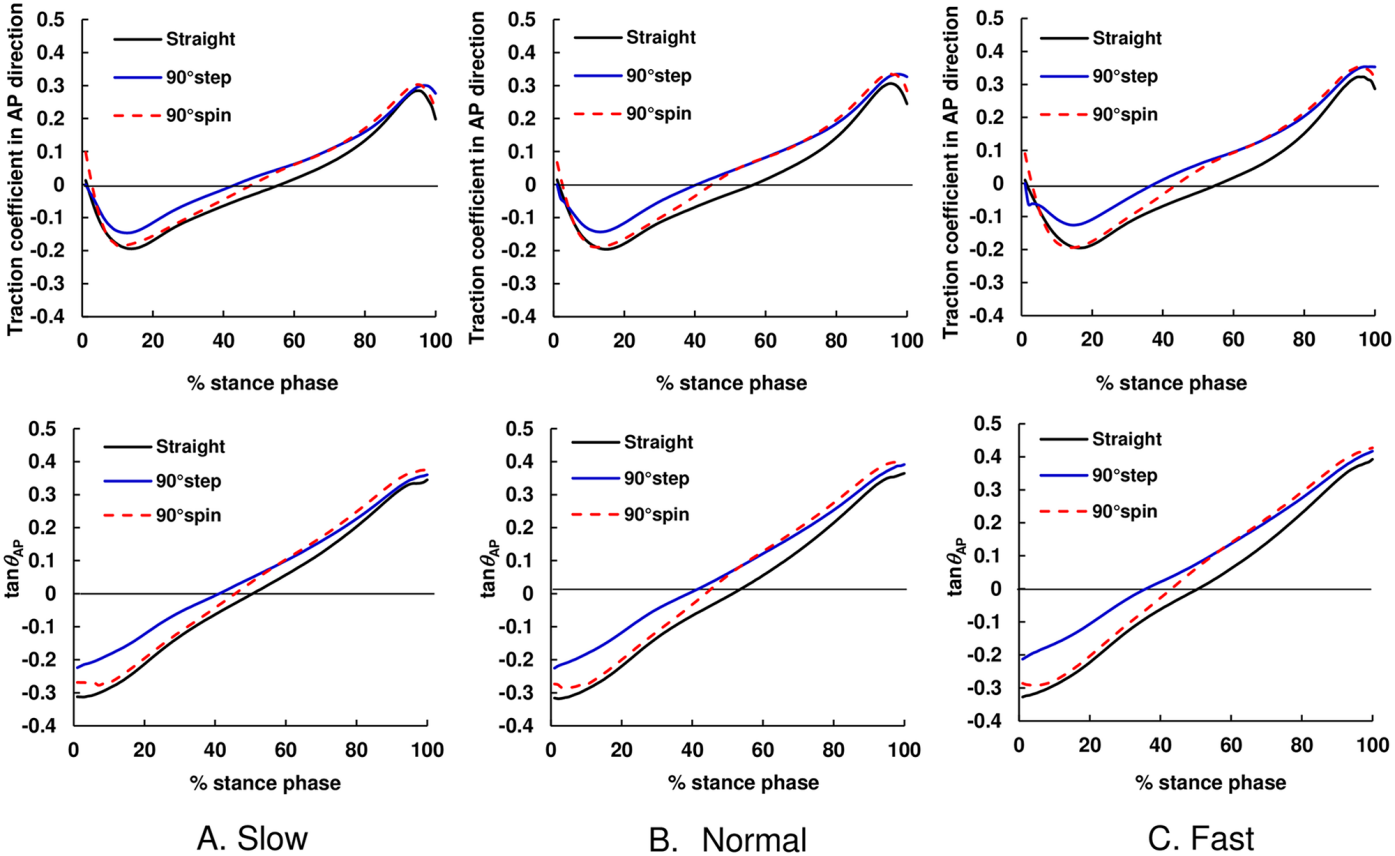
The average temporal pattern of mean values of traction coefficient and tangent of COM–COP angle in the AP direction during straight walking and turning. The black solid line shows straight walking, blue solid line shows 90° step turns, and red dashed line shows 90° spin turns. (A) Slow, (B) Normal, and (C) Fast.

**Fig 6:**
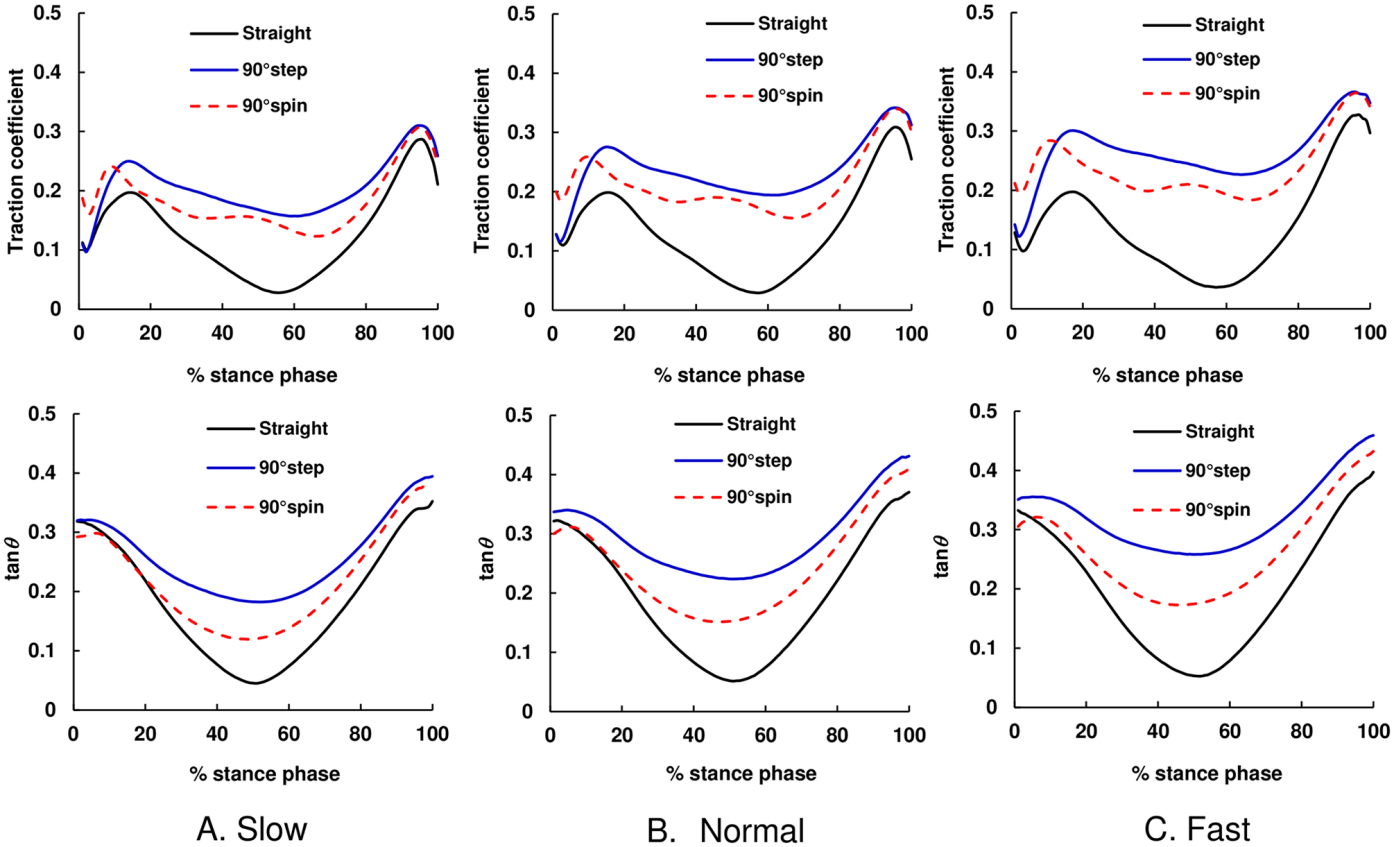
The average temporal pattern of mean values of the resultant traction coefficient and tangent of COM–COP angle during straight walking and turning. The black solid line shows straight walking, blue solid line shows 90° step turns, and red dashed line shows 90° spin turns. (A) Slow, (B) Normal, and (C) Fast.

As shown in Fig 4, the magnitude of the traction coefficient and COM–COP angle tangent in the ML direction for turning trials were higher than those in straight walking trials during almost the whole stance phase for all speed conditions. As shown in Fig 5, the magnitude of the traction coefficient and COM–COP angle tangent in the AP direction for turning trials tended to be smaller than those for straight walking trials during the braking phase (approximately 0%–50% of the stance phase) but larger than those for the propulsion phase (approximately 50%–100% stance phase). However, the difference in the traction coefficients and COM–COP angle tangents in the AP direction among trial types was not so significant compared with the ML direction (see Fig 4). As shown in Fig 6, the difference in both traction coefficient and tan*θ* between straight walking trials and turning trials was significant around the mid-stance phase for each speed condition and that for step turns tended to be larger than that for spin turns.

### Required coefficient of friction in the ML and AP directions

**Fig 7:**
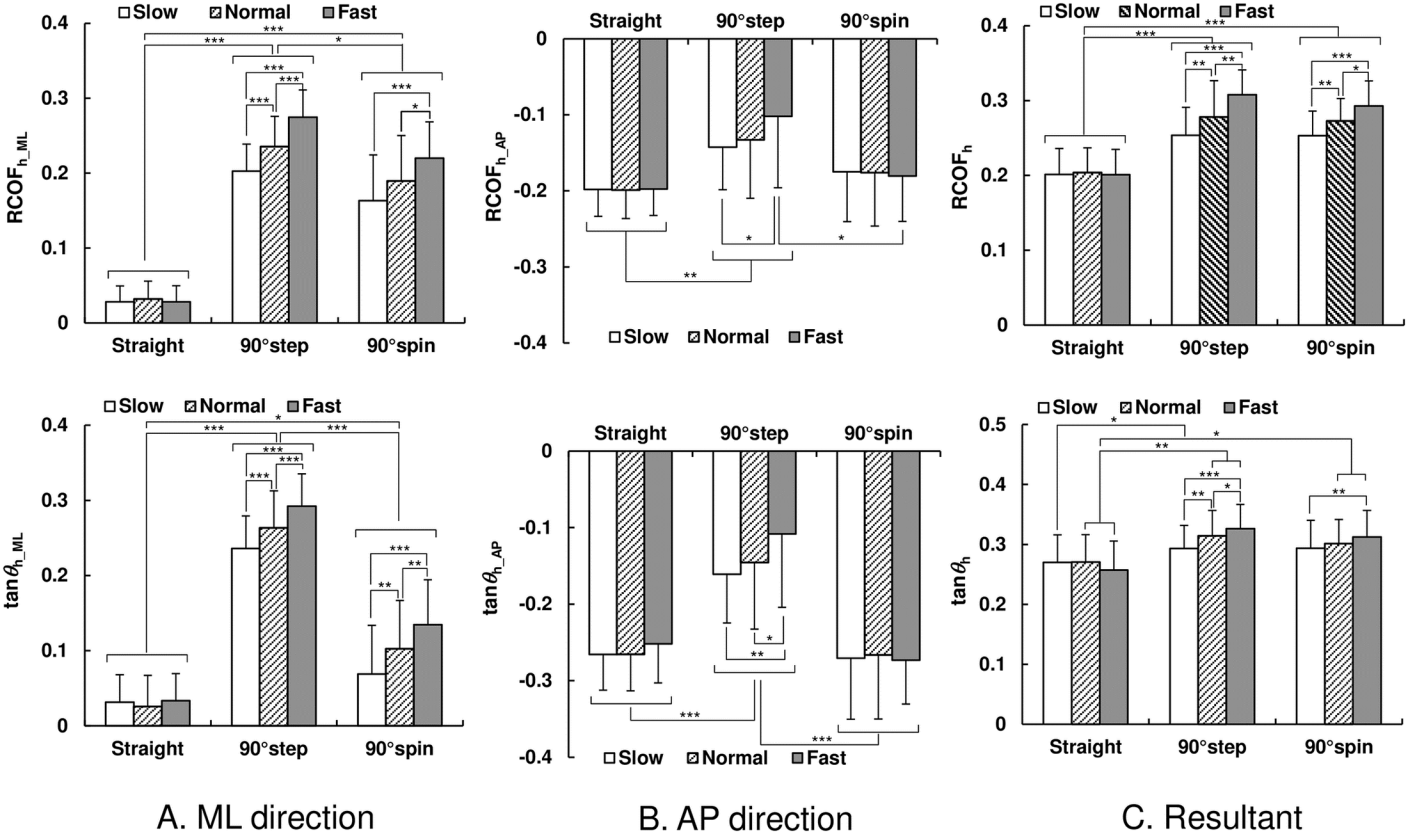
The mean value of required coefficient of friction and tangent of COM–COP angle during the weight acceptance phase. Error bars represent standard deviation. (A) ML direction; (B) AP direction; (C) Resultant: * p < 0.05, ** p < 0.01, and *** p < 0.001.

As shown in Fig 7A, the mean values of RCOF_h_ML_ and tan*θ*_h_ML_ for step and spin turns were significantly higher than those for straight walking (p < 0.05) and increased with an increase in walking speed (p < 0.05). Those values for step turn were significantly higher than those for spin turn irrespective of walking speed conditions (p < 0.05). As shown in Fig 7B, the mean values of RCOF_h_AP_ and tan*θ*_h_AP_ for straight walking and spin turn trials were not significantly affected by walking speed (p > 0.05). Those for step turns tended to be smaller than those for straight walking and spin turns. As shown in Fig 7C, the mean values of RCOF_h_ for turning trials were higher than those for straight walking and increased with an increase in walking speed (p < 0.05). The mean values of tan*θ*_h_ tended to increase with an increase in walking speed (p < 0.05 for step turns; p < 0.05 for spin turns in the slow and fast speed conditions) and those for turning trials were higher than those for straight walking trials at normal and fast speeds.

**Fig 8:**
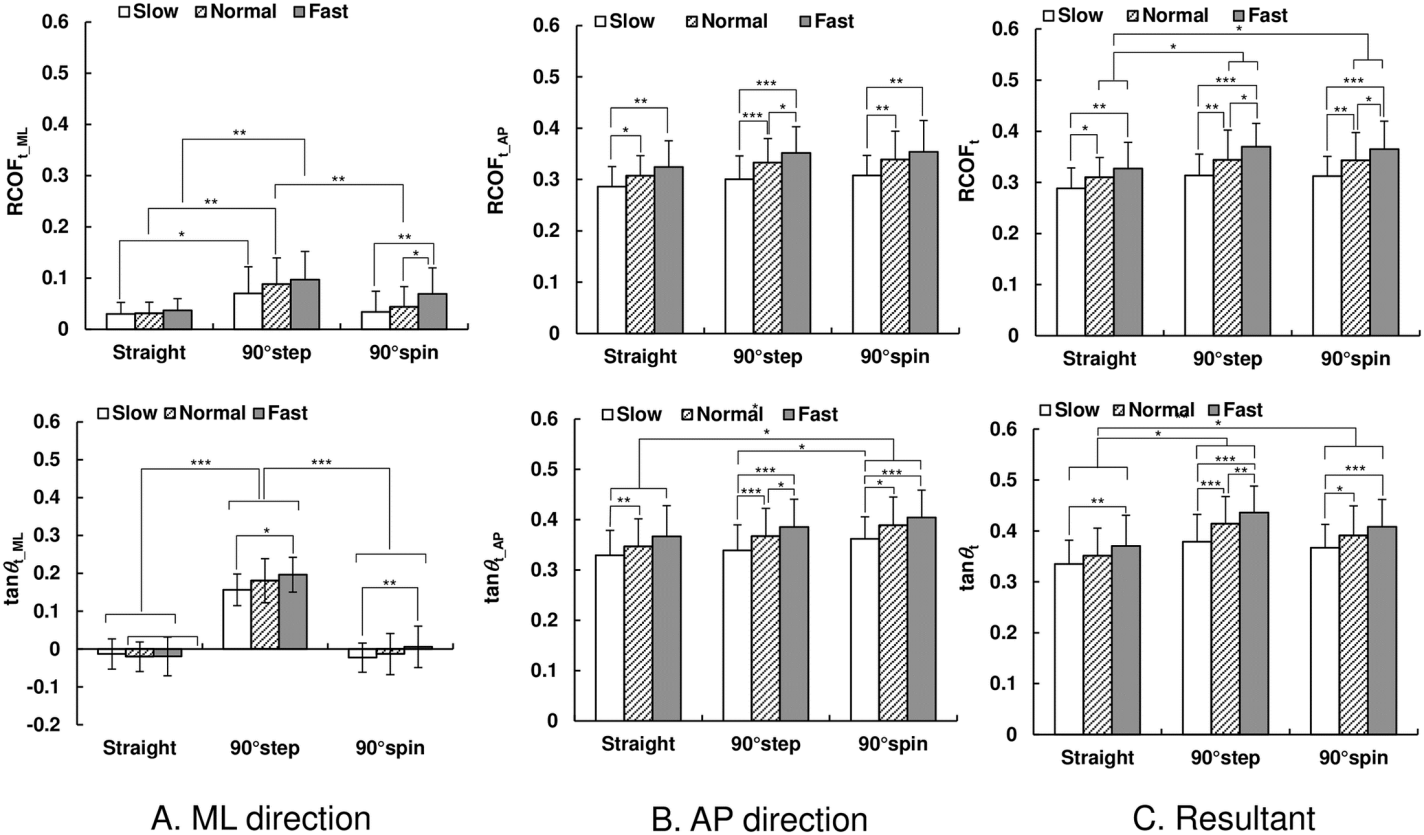
The mean value of required coefficient of friction and tangent of COM–COP angle during the toe-off phase. Error bars represent standard deviation. (A) ML direction; (B) AP direction; (C) Resultant: * p < 0.05, ** p < 0.01, and *** p < 0.001.

As shown in Fig 8A, the mean values of RCOF_t_ML_ and tan*θ*_t_ML_ for step turns were higher than those for straight walking (p < 0.05), and those of spin turns tended to be lower than those of step turns. As shown in Fig 8B, there were no differences in the mean values of RCOF_t_AP_ between trial types (p >0.05); however, they did increase with an increase in walking speed (p < 0.05). On the other hand, the mean values of tan*θ*_t_AP_ for spin turn trials were higher than those for straight walking (p < 0.05). The mean values of tan*θ*_t_AP_ for turning trials at slow speeds were smaller than those for normal and fast speeds (p < 0.05). As shown in Fig 8C, at normal and fast speeds, the mean values of RCOF_t_ were higher than those for straight walking trials, and these values increased for turning trials with increased walking speed (p < 0.05). The same trend can be seen for the mean values of tan*θ*_t_.

The bivariate regression analysis demonstrated that the mean values of RCOF-related parameters and tan*θ*-related parameters strongly correlated with each other (R = 0.87, p < 0.01 for tan*θ*_h_ML_ vs. RCOF_h_ML_; R = 0.94, p < 0.001 for tan*θ*_h_AP_ vs. RCOF_h_AP_; R = 0.98, p < 0.001 for tan*θ*_h_vs. RCOF_h_; R = 0.91, p < 0.001 for tan*θ*_t_ML_ vs. RCOF_t_ML_; R = 0.95, p < 0.001 for tan*θ*_t_AP_ vs. RCOF_t_AP_; and R = 0.94, p < 0.001 for tan*θ*_t_ vs. RCOF_t_). Bivariate regression analysis between centripetal force and RCOF values for turning trials in the ML direction revealed a strong positive correlation between the two values (R = 0.97, p < 0.01 for RCOF_h_ML_ vs. *F*_c_ and R = 0.95, p < 0.01 for RCOF_t_ML_ and *F*_c_).

## Discussion

Our results demonstrated that the magnitude of the traction coefficient and COM–COP angle tangent in the ML direction for turning trials were higher than those in straight walking trials during almost the whole stance phase for all speed conditions (Fig 4). The results also indicated that, during weight acceptance phase, the RCOF_h_ML_ and tan*θ*_h_ML_ values for step and spin turns were larger than those during straight walking (Fig 7). The increased RCOF_h_ML_ values contributed to the increased resultant RCOF_h_ values during step and spin turns (Fig 7C). During push-off phase, RCOF_t_ML_ and tan*θ*_t_ML_ values for step turns were higher than those for straight walking, which resulted in the increase in the RCOF_t_ values (Fig 8). These results support our first hypothesis. The RCOF_h_ML_ and tan*θ*_h_ML_ for step and spin turns increased with an increase in walking speed (Fig 7), which was due to the increase in centripetal force; there were strong positive correlation between RCOF_h_ML_ values and the centripetal force. On the other hand, during the push-off phase, RCOF_t_ML_ and tan*θ*_t_ML_ values were not affected significantly by walking speed but RCOF_t_AP_ and tan*θ*_t_AP_ values increased with increasing walking speed [Fig 8A and 8B]. These results partially support our second hypothesis.

As we expected, the tan*θ*_h_ML_ values for turning trials were larger than those for straight walking and they increased with walking speed [Fig 7A], which would be due to increased centripetal force. This trend was also observed for tan*θ*_t_ML_ for step turn trials but not for spin turn trials. During the push-off phase during spin turns, as shown in Fig 3, the pelvis rotation angle was almost −90°, indicating that the rotation of the body was almost finished and COM travels in the AP direction. The increased tan*θ*_t_AP_ with walking speed was possibly due to the increase in step length; thus, the RCOF_t_AP_ increased with an increase in walking speed [Fig 8B]. On the other hand, during the push-off phase during step turns, the pelvis rotation angle was still −60°, which indicates that the centrifugal force still acts on COM in this instance. As shown in Fig 7B, the magnitudes of RCOF_h_AP_ and tan*θ*_h_AP_ values for step turns were lower than those for straight walking and spin turn. For step turns, the COM is inside base of support (BOS) during the weight acceptance phase, whereas it is outside BOS for spin turn and near the border of BOS for straight walking [21]; thus, the distance between COM and COP during the weight acceptance phase in the AP direction for step turns was shorter than that for straight walking and spin turns, which resulted in smaller tan*θ*_h_AP_ and RCOF_h_AP_.

Similar to our current study, Fino and Lockhard [13] demonstrated that there were no significant differences in the resultant RCOF values between step and spin turns. Whether or not slip occurs is evaluated by the relationship between the resultant RCOF value and the friction coefficients at the shoe–floor interface, and not just by the ML or AP components of the RCOF values. However, even if the magnitude of RCOF value is the same, the direction of slipping will be affected by the by the ratio of ML and AP component of RCOF values. The results in the current study demonstrated that the RCOF_h_ML_ and tan*θ*_h_ML_ values for step turns were significantly higher than those for spin turns at all walking speed conditions [Fig 7A]. Furthermore, the contribution of RCOF_h_ML_ and tan*θ*_h_ML_ values to the resultant RCOF_h_ and tan*θ*_h_ values was significant during step turns compared with spin turns. These findings support our third hypothesis. We also found that the centripetal force during step turns was larger than that during spin turns (Table 2), which was caused mainly by the shorter turning radius of step turns. This larger centripetal force could cause the difference in tan*θ*_h_ML_ and RCOF_h_ML_ between step and spin turns at each speed condition [Fig 7A]. On the basis of these results, when slip occurs, step turns will cause slips more laterally than spin turns.

There were significant differences in traction coefficient values around the mid-stance phase between straight walking and turn trials. As shown in Fig 5, the traction coefficient for straight walking and turning in the AP direction was almost zero around the mid-stance phase because in the sagittal plane during this phase, COM is almost right over COP, which means tan*θ*_AP_ was almost zero. Thus, the differences in the traction coefficient during the mid-stance phase between straight walking and turning were caused by the difference in the traction coefficient in the ML direction. This means that during the mid-stance phase during turning, tangential force to floor surface is mainly applied in the ML direction, and slip occurs in the lateral direction if the traction coefficient reaches the static coefficient of friction between footwear and floor.

In the current study, the RCOF_h_ value was defined as the maximum peak value of the traction coefficient between 5% and 50% of the stance phase. We confirmed that the RCOF_h_ values, as determined using the criterion proposed by Chang et al. [25], were not different from those determined using our criterion in straight walking. In the criterion proposed by Chang et al. [25], the longitudinal GRF (in the AP direction) must be applied backward at the RCOF instance because RCOF values are mainly determined with shear force in the AP direction during straight walking. However, during turning, transverse shear force (in the ML direction) is not negligible and substantially contributes to the RCOF values. Therefore, it is unclear whether the criterion used in the literature can be applied to the determination of RCOF values for turning gait; further investigation is needed.

A limitation of the gait trials was that step length may be regulated by the force plate configuration. During straight walking, if the walking speed is increased with increasing step length, the walking speed increases the COM–COP angles, resulting in increased RCOF values. However, in the current study, the subjects regulated the walking speed mainly by varying cadence. Therefore, the RCOF values were not significantly affected by walking speed in straight walking. In turning trials, the subjects were asked to turn with the left foot as the pivot foot for step turn and to turn with the right foot as the pivot foot for spin turn. Chang et al. [26] found that the RCOF values of both feet could be different for the same individual. The RCOF of both feet during step and spin turns could also be different due to the foot dependence. Further studies are needed to investigate the effect of foot dependence on the RCOF values during spin and step turns. As another limitation, we only studied 16 young adults. Akram et al. [27] found that healthy older adults demonstrated a preference for spin turns, whereas healthy young adults preferred step turns, which is a more stable [28] and biomechanically efficient turning strategy [21]. In the future, it will be necessary to examine RCOF values during turning for older adults because older adults often have difficulty in maintaining lateral postural stability [29, 30] and lateral falls do cause life-threatening injuries in older people [31]. Older adults walk with reduced walking speed and shorter step length compared with young adults [32, 33]. Therefore, it is assumed that older adults exhibit slower turning speed and shorter turning radius during turning compared with young adults. Therefore, the centripetal force during turning will be different between young and older adults, which affects the respective RCOF values in the ML direction. However, further investigation is needed for clarification.

## Conclusions

This study was the first attempt to investigate the RCOF values and COM–COP angle tangent in the ML and AP directions while straight walking and turning. The results of the present study indicate that the RCOF values and COM–COP angle tangent in the ML direction during turning at weight acceptance phase were higher than those during straight walking, and those values increased with increasing walking speed. The increased RCOF values in the ML direction contributed to the increase in the resultant RCOF values during turning. We also found that the ML component of the RCOF and COM–COP tangent values during weight acceptance for step turns were higher than those for spin turns, whereas there were no significant differences in the resultant RCOF and COM–COP tangent values between step and spin turns. The centripetal force had strong positive correlation with the ML component of the RCOF and COM–COP tangent values. Thus, turning is likely to cause lateral slip at weight acceptance because of the increased centripetal force compared with straight walking. Step turns with high speed are more likely to result in the slip in the lateral direction. The results of the current study suggest that the ML and AP components of the RCOF values and the resultant RCOF values should be taken into account to further the understanding of how gait prevents slips and falls during turning. Future work should test at-risk population and compare with the present results.

## Supporting information

S1 Dataset Approaching speed. (XLSX)

S2 Dataset Turning radius, turning speed, and centripetal force. (XLSX)

S3 Dataset Peak required coefficient of friction and tangent of COM–COP angle during the weight acceptance. (XLSX)

S4 Dataset Peak required coefficient of friction and tangent of COM–COP angle during the toe-off phase. (XLSX)
